# Inhibition of *Streptococcus* Biofilm Formation by 6′-Sialyllactose and *N*-Acetylneuraminic Acid

**DOI:** 10.3390/dj14010041

**Published:** 2026-01-07

**Authors:** Yohei Sato, Yuta Watanabe, Tatsuhiro Ayabe, Takeshi Kokubo

**Affiliations:** Institute of Health Sciences, Kirin Holdings Co., Ltd., 2-26-1-12-12 Muraoka-Higashi, Fujisawa 251-8555, Japan; yuta_watanabe@kirin.co.jp (Y.W.); tatsuhiro_ayabe@kirin.co.jp (T.A.); takeshi_kokubo@kirin.co.jp (T.K.)

**Keywords:** biofilm, oral microbiome, *Streptococcus mutans*, human milk oligosaccharides, dentistry, microbiology

## Abstract

**Background/Objectives**: Oral hygiene is crucial for maintaining overall health, as poor oral care can lead to various systemic diseases. Although xylitol is widely used to inhibit plaque formation, more effective agents are needed to control oral biofilms. Herein, we evaluated the inhibitory effects of sialyllactose (SL), a type of human milk oligosaccharide (HMO), and its partial structure *N*-acetylneuraminic acid (Neu5Ac) against *Streptococcus* biofilm. **Methods**: Under a CO_2_ atmosphere, *Streptococcus mutans* and mixed *Streptococcus* species were each cultivated in vitro, and the inhibitory effects of HMOs [2′-fucosyllactose, 3′-sialyllactose (3′-SL) and 6′-sialyllactose (6′-SL)] and Neu5Ac on biofilm formation were evaluated. Bacterial biofilm formation was quantified using the crystal violet assay. Biofilm architecture and viability were visualized using confocal laser-scanning microscopy (CLSM) with SYTO9/propidium iodide staining. Transcriptomic responses of *S. mutans* biofilms to the test compounds were analyzed by RNA-Seq. Statistical analysis was performed using one-way analysis of variance followed by Tukey’s test. **Results**: SLs and Neu5Ac at 100 mM significantly inhibited *S. mutans* biofilm formation, with stronger effects than those of xylitol. The inhibitory effects varied among HMOs, with 6′-SL being more effective than 3′-SL and Neu5Ac being most effective. These effects were consistent in assays targeting biofilms formed by other *S. mutans* strains and in a mixed biofilm comprising *Streptococcus* species. Gene expression analysis suggested that the inhibitory mechanism involves the physical inhibition of surface adhesion and stress-induced regulation of gene expression. **Conclusions**: This study provides insights into the physiological significance of HMOs in the oral cavities of humans. HMOs exhibited potential as functional foods to control oral biofilm formation and reduce the risk of oral and systemic diseases.

## 1. Introduction

More than 700 bacterial species and 1 × 10^8^–1 × 10^9^ bacterial cells/mL saliva inhabit the human oral cavity, wherein a healthy oral microbiome helps prevent the invasion of pathogenic bacteria and viruses [1,2]. Some oral bacteria metabolize dietary sugars and form film-like structures called biofilms that adhere strongly to the surfaces of teeth and alveolar ridges. Within these biofilms, bacteria proliferate and gain protection from antibacterial agents and phagocytic cells [3]. They also upregulate virulence-associated genes through quorum sensing, thereby producing acids and other harmful substances [3,4]. Consequently, bacteria can damage teeth and alveolar ridges, causing inflammation that leads to oral diseases such as dental caries and periodontal disease. Biofilms also promote the growth of highly pathogenic bacteria, increasing the risk of systemic diseases such as arteriosclerosis and diabetes [2,5,6]. Moreover, individuals with an increased abundance of *Fusobacterium nucleatum* in their oral biofilms have been reported to exhibit a heightened risk of colon cancer [7,8]. Taken together, controlling oral biofilms is important for maintaining overall human health.

Physical approaches such as tooth brushing and ultrasonic tooth cleaning, as well as biochemical approaches such as dental antiseptics, have been developed to control and remove biofilms [9,10,11,12,13,14,15]. These conventional mechanical and chemical measures have practical and biological limitations: patient adherence to daily mechanical plaque control is often variable, professional interventions are intermittent, and chemical agents can have limited substantivity or undesirable side effects, which together restrict long-term maintenance of oral biofilm control [16,17]. Emerging adjunctive modalities, such as ozone therapy and photobiomodulation (including antimicrobial photodynamic therapy), are being investigated to complement existing strategies by directly reducing biofilm microbial load or modulating host inflammatory and wound-healing responses [18,19]; nevertheless, these device- and parameter-dependent methods require protocol-specific validation for efficacy, safety (e.g., ozone inhalation risk; dose-dependent effects of photobiomodulation), and effects on the resident oral microbiome. Consequently, while dental treatment combined with effective self-care remains the most practical means of plaque control, the range of measures suitable for routine patient self-care is limited. Sugar alcohols, including xylitol and erythritol, have been explored for plaque control and are widely used as sugar substitutes owing to their sweetness and non-cariogenic properties. However, these sugar alcohol solutions raise concerns because of their potential effects on oral microbiome diversity and balance [20], indicating the need for novel biofilm-inhibitory strategies with different mechanisms of action.

Human milk oligosaccharides (HMOs) are complex oligosaccharides found in human milk and represent the third-most common solid component in human milk. HMOs consist of a core lactose unit with additional monosaccharides, such as glucose, galactose, *N*-acetylglucosamine, fucose, and *N*-acetylneuraminic acid (Neu5Ac). More than 200 structural varieties of HMOs have been identified [21,22]. Among them, 2′-fucosyllactose (2′-FL), 3′-sialyllactose (3′-SL), and 6′-sialyllactose (6′-SL), the smallest HMOs, are well studied in the field of infant nutrition. HMOs (individual and mixed) extracted from human milk have been reported to inhibit the growth and biofilm formation of group B *Streptococcus*, which forms biofilms in the birth canal and causes neonatal meningitis [23,24,25,26]. Furthermore, Faria et al. found that 3′-SL inhibits biofilm formation by *Streptococcus mutans*, a causative species of dental caries [27], supporting the emerging functional capabilities of HMOs against biofilms. However, the inhibitory effects of other HMOs on oral bacterial biofilms, as well as the impact of structural variation among HMOs, have not been fully considered.

This study evaluated the antibiofilm effects of HMOs as a practical approach to oral biofilm control through experiments using multiple *S. mutans* strains and a mixture of oral *Streptococcus* strains. The null hypothesis of this study is that HMOs do not significantly affect biofilm formation by oral *Streptococcus* species. Our findings deepen the understanding of the antibiofilm actions of HMOs and may contribute to the development of new solutions that overcome the limitations of conventional oral biofilm-control methods.

## 2. Materials and Methods

### 2.1. Chemical Reagents and Bacterial Strains

Unless otherwise stated, all chemical reagents were obtained from Fujifilm Wako Pure Chemical Corporation (Osaka, Japan). HMOs (2′-FL, 3′-SL, and 6′-SL) and Neu5Ac, biosynthesized via bacterial fermentation, were obtained from Kyowa Hakko Bio Co., Ltd. (Tokyo, Japan). *S. mutans* strains (*S. mutans* UA159, 25172, and 35668) were obtained from the American Type Culture Collection (Manassas, VA, USA). Other *Streptococcus* strains (*S. sanguinis* 5708, *S. mitis* 12971, *S. gordonii* 12995, *S. intermedius* 12996, and *S. oralis* 12997) were provided by the Japan Collection of Microorganisms, RIKEN BRC, which is participating in the National BioResource Project of MEXT, Japan.

### 2.2. Culture and Quantification of Biofilms (Crystal Violet Assay)

Before inoculation into culture plates for biofilm formation assays, brain heart infusion broth (BHI; Difco Laboratories, Detroit, MI, USA) was used to grow *Streptococcus* strains in an aerobic atmosphere containing 5% CO_2_ (AnaeroPack-CO_2_; Mitsubishi Gas Chemical Co., Ltd., Tokyo, Japan) at 37 °C. For the biofilm formation assay, 1% sucrose-added BHI (BHIs) was prepared because sucrose is reported to promote biofilm formation [28]. Test compounds (xylitol, 2′-FL, 3′-SL, 6′-SL, and Neu5Ac) were prepared as 200 mM stock solutions in BHIs.

To evaluate *S. mutans* biofilm formation, 90 μL of a bacterial cell suspension adjusted to an optical density at 600 nm (OD_600_) of 0.02 in BHIs were mixed with 90 μL of each test compound added to BHIs in 96-well microtiter plates (final working concentration of each test compound was 100 mM). The negative control (NC) comprised BHIs without test compounds. The 100 mM working concentration was chosen based on preliminary experiments showing strong inhibition of early biofilm formation within 6 h, whereas no effect was observed at 24 h, suggesting that the compounds act during the initial adhesion phase.

To evaluate biofilm formation by *Streptococcus* species, bacterial suspensions of *S. mutans* UA159, *S. sanguinis* 5708, *S. mitis* 12971, *S. gordonii* 12995, *S. intermedius* 12996, and *S. oralis* 12997 were mixed with the same number of cells, adjusted to a final OD_600_ of 0.02 in BHIs, and then mixed with BHIs containing test compounds as previously mentioned.

After static incubation for 6 h in an aerobic atmosphere containing 5% CO_2_ at 37 °C, each well was washed twice with 200 μL of pure water and filled with 200 μL of 0.1% crystal violet solution to stain the biofilm. After 15 min of staining, the plates were washed twice, and 200 μL of 99.5% ethanol were added to extract the crystal violet. After 1 h, extracts were mixed with a pipette. The entire extracts were transferred to a new plate, and their absorbance at 590 nm was measured using SpectraMax M3 (Molecular Devices, LLC., San Jose, CA, USA).

### 2.3. Confocal Laser-Scanning Microscopy

The BacLight LIVE/DEAD bacterial viability kit (Thermo Fisher Scientific Inc., Waltham, MA, USA) was used to detect bacterial viability and visualize biofilms formed by *S. mutans* UA159 using CLSM (LSM980; Carl Zeiss AG, Oberkochen, Germany) [29]. The 6 h biofilms formed on 35 mm glass-bottom dishes (AGC Techno Glass Co., Ltd., Shizuoka, Japan) were stained with 2.5 μM SYTO9 and 2.5 μM propidium iodide (PI) for 15 min following the manufacturer’s protocol. Live bacteria were stained green (excitation/emission channels were 483/500 nm), while dead bacteria and extracellular DNA were stained red (excitation/emission channels were 305/617 nm). Images of three randomly selected points on each sample were obtained using a ×20 objective lens. The observed total volume of stained areas was calculated using Imaris 10.2.0 (Oxford Instruments plc., Abingdon, UK).

### 2.4. RNA Sequencing (RNA-Seq) and Analysis

Bacteria in biofilms were collected from each well into 2 mL tubes via scraping with a pipette tip followed by pipetting. Before extraction, RNA was stabilized with a twofold volume of RNAprotect Bacteria Reagent (Qiagen, Hilden, Germany). The bacteria were then centrifuged (5000× *g*, 4 °C, 10 min), the supernatant was discarded, and the bacteria were treated with 200 μL of Tris-EDTA buffer (pH 8.0, Sigma-Aldrich Inc., St. Louis, MO, USA) containing 1 mg/mL lysozyme and >60 mAU/mL proteinase K (Qiagen) at 22 °C and 500 rpm for 10 min. Spheroplasts of bacteria were resuspended in 700 μL of RLT buffer (Qiagen) containing 40 mM dithiothreitol. After adding 500 μL of 99.5% ethanol, total RNA was purified using an RNeasy Mini Kit (Qiagen) following the manufacturer’s protocol.

RNA-Seq was outsourced to Takara Bio Inc. (Kusatsu, Japan). Stranded RNA libraries were prepared using the Stranded Total RNA Prep, Ligation with Ribo-Zero Plus Kit (Illumina, San Diego, CA, USA). Paired-end 2 × 150 bp sequencing was performed on a NovaSeq 6000 sequencer (Illumina). After trimming and filtering using fastp (version 0.23.2) with default parameters to remove low-quality reads, RNA-Seq reads were mapped to the *S. mutans* genome accession GCF_000007465.2 using STAR (version 2.7.10a) with default parameters. Based on RefSeq Gene ID, each gene count was determined using featureCounts (version 2.0.1) [30]. The normalized levels of genes, represented by the trimmed mean of M-values, were generated with the edgeR [31] package in R (version 4.2.2).

### 2.5. Statistics

All data were obtained in triplicate for each experiment and expressed as the mean and standard deviation (SD). Statistical analysis was performed using the Shapiro–Wilk normality test and one-way analysis of variance followed by Tukey’s post hoc test for multiple comparisons, using BellCurve for Excel (version 4.07; Social Survey Research Information Co., Ltd., Tokyo, Japan). A *p* value of <0.05 was considered significant.

## 3. Results

### 3.1. SLs and Neu5Ac Inhibited Biofilm Formation by Streptococcus

**Figure 1:**
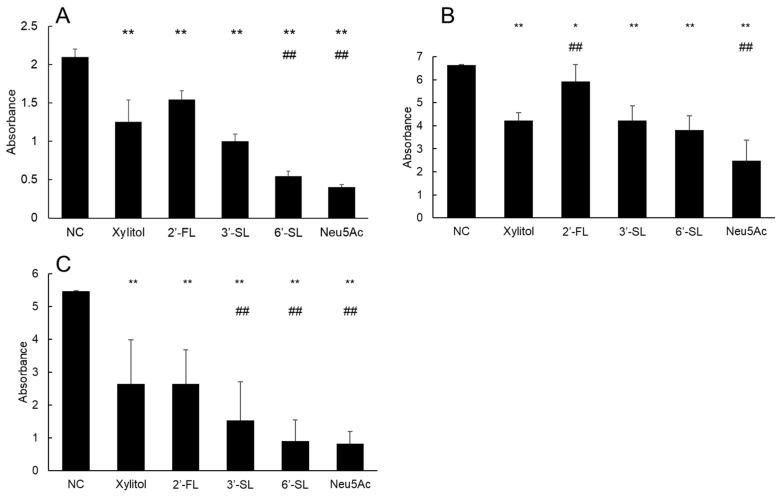
Quantification of biofilm formation by *S. mutans* UA159 (**A**), 25175 (**B**), and 35668 (**C**) after 6 h of incubation. *: *p* < 0.05, **: *p* < 0.01 versus NC group. ##: *p* < 0.01 versus xylitol group. NC = negative control, 3′-SL = 3′-sialyllactose, 6′-SL = 6′-sialyllactose, Neu5Ac = *N*-acetylneuraminic acid.

After 6 h of incubation of three *S. mutans* strains, solid biofilms were formed on the bottom of the microtiter plates. Compared with the findings in the NC group, all test compounds decreased the absorbance of crystal violet (Table 1, Figure 1). For *S. mutans* UA159 and 35668, absorbance was significantly lower in the 6′-SL and Neu5Ac groups than in the xylitol group (both *p* < 0.01), indicating stronger inhibitory effects of 6′-SL and Neu5Ac. For *S. mutans* 25175, Neu5Ac significantly decreased absorbance compared to xylitol (*p* < 0.01), whereas 6′-SL showed no significant effect (*p* = 0.20). Additionally, 2′-FL had weaker effects on the absorbance of *S. mutans* 25175 than xylitol (*p* < 0.01). For *S. mutans* 35668, 3′-SL significantly decreased absorbance compared to xylitol (*p* < 0.01).

**Figure 2:**
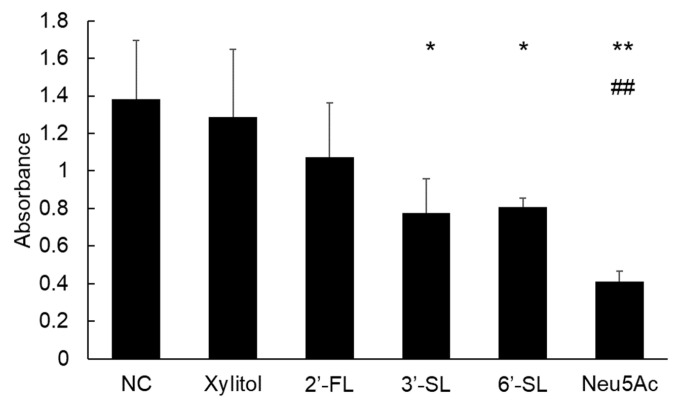
Quantification of biofilm formation by a mixture of *Streptococcus* strains after 6 h of incubation. *: *p* < 0.05, **: *p* < 0.01 versus NC group. ##: *p* < 0.01 versus xylitol group. NC = negative control, 3′-SL = 3′-sialyllactose, 6′-SL = 6′-sialyllactose, Neu5Ac = *N*-acetylneuraminic acid.

Unlike the experiments of single *S. mutans* strain, xylitol and 2′-FL did not decrease the absorbance of the mixed *Streptococcus* culture (*p* = 0.58), whereas SLs and Neu5Ac significantly decreased absorbance (Figure 2; *p* = 0.02 for 3′-SL, *p* = 0.03 for 6′-SL, *p* < 0.01 for Neu5Ac).

### 3.2. LIVE/DEAD Assay Revealed Compositional Changes in the Biofilm Matrix

**Figure 3:**
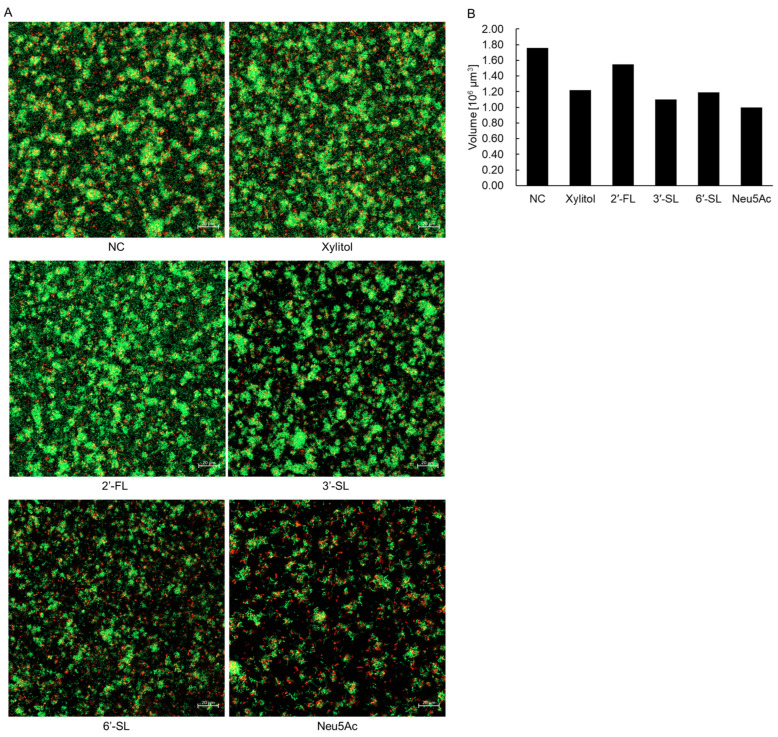
CLSM analysis of biofilm formation by *S. mutans* UA159. (**A**) Biofilms stained using the BacLight LIVE/DEAD bacterial viability kit. Green areas indicate nucleic acids in live cells, and red areas indicate nucleic acids in dead cells. (**B**) The total volume of the stained biofilm was calculated using Imaris software. NC = negative control, 3′-SL = 3′-sialyllactose, 6′-SL = 6′-sialyllactose, Neu5Ac = *N*-acetylneuraminic acid.

Using *S. mutans* UA159 as a representative strain, biofilm structure was observed using SYTO9/PI staining (Figure 3A). In the NC group, the glass bottom of the culture plate was filled with large clumps of live bacteria. Adding xylitol or 2′-FL had minimal effects on biofilm structure. In contrast, 6′-SL and Neu5Ac significantly reduced the size and number of bacterial clumps. The total volume of stained areas was slightly decreased in the 2′-FL, xylitol, 3′-SL, 6′-SL, and Neu5Ac groups (Figure 3B).

### 3.3. SLs and Neu5Ac Induced Stress-Responsive Gene Expression and Suppressed Bacteriocin Gene Expression in S. mutans

**Figure 4:**
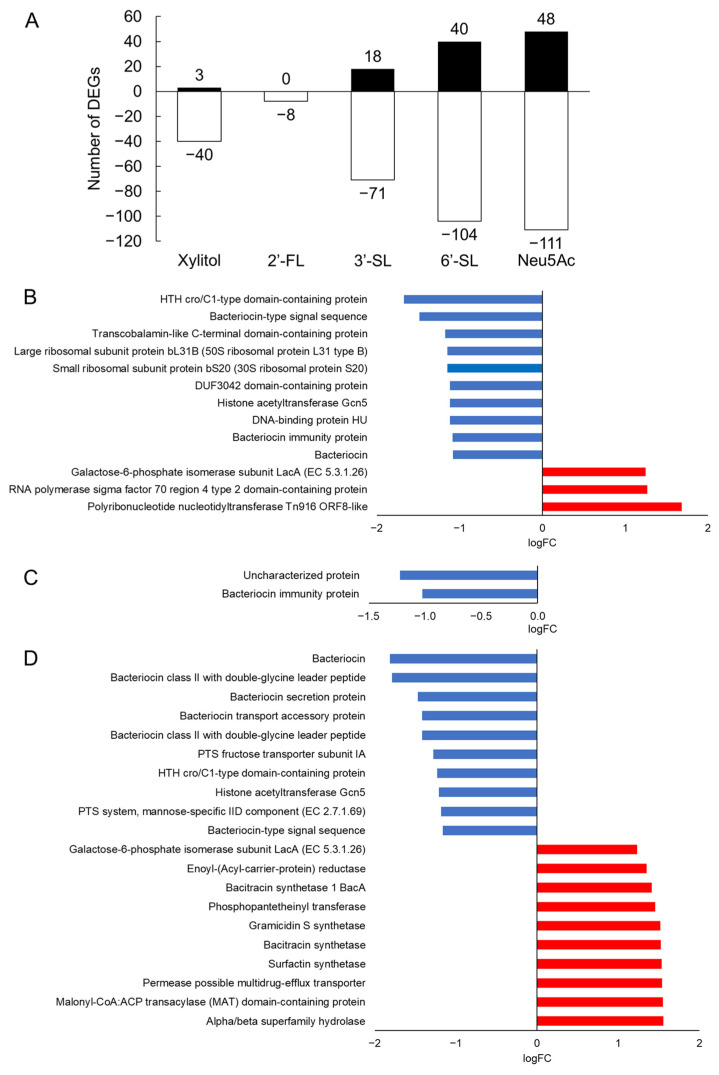

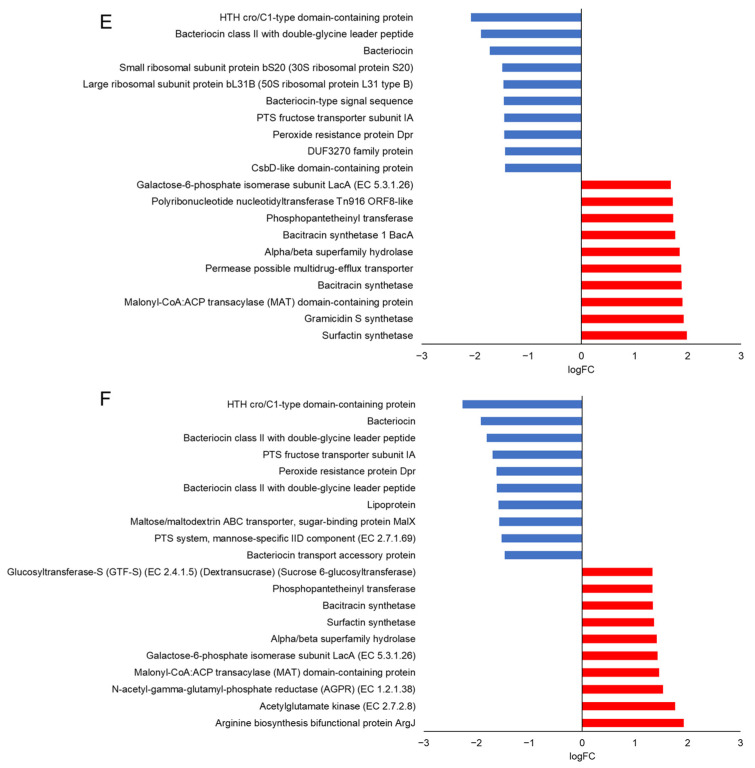
Differential gene expression and functional annotation in response to the test compounds. (**A**) Number of DEGs in response to xylitol, 2′-FL, 3′-SL, 6′-SL, and Neu5Ac exposure compared with the effect of the NC. Black bars indicate upregulated genes, and white bars indicate downregulated genes. (**B**–**F**) Functional annotation of DEGs for each group: (**B**) xylitol, (**C**) 2′-FL, (**D**) 3′-SL, (**E**) 6′-SL, and (**F**) Neu5Ac. Blue bars represent downregulated gene categories, and red bars represent upregulated gene categories. The x-axis presents log fold change (logFC). The top 10 gene categories with the highest degree of upregulation (logFC > 1; red bars) and downregulation (logFC < −1; blue bars) are listed. NC = negative control, 3′-SL = 3′-sialyllactose, 6′-SL = 6′-sialyllactose, Neu5Ac = *N*-acetylneuraminic acid.

RNA-Seq analysis of *S. mutans* UA159 biofilms revealed changes in gene expression between each test compound group and the NC group. Relatively few changes were observed in the xylitol [43 differentially expressed genes (DEGs)] and 2′-FL groups (8 DEGs), whereas 3′-SL (89 DEGs), 6′-SL (144 DEGs), and Neu5Ac (159 DEGs) more strongly affected gene expression in *S. mutans* (Figure 4 and Figure S1). Xylitol suppressed the expression of universal genes, including ribosomal protein and histone acetyltransferase (Figure 4B). In contrast, SLs and Neu5Ac upregulated stress-responsive gene clusters (such as *mub* and *arg*) and suppressed bacteriocin-related gene expression (Figure 4D–F).

## 4. Discussion

In this study, we investigated the inhibitory effects of HMOs and Neu5Ac against biofilm formation by *Streptococcus* species. Compared with xylitol, which is well known for its plaque-inhibitory properties, we found for the first time that SLs, a class of HMOs, and their partial structure Neu5Ac inhibited biofilm formation more strongly. The strength of this inhibited varied depending on the HMO type, with 6′-SL displaying stronger effects than 3′-SL, and Neu5Ac exhibiting even stronger effects than the SLs. We assessed the effects of SLs and Neu5Ac on biofilms formed by bacteria cultured for 6 h (Figure 1 and Figure 2). However, in our preliminary experiments, no inhibitory effects were observed in 24 h biofilms for any treatment, including xylitol. Therefore, it is likely that the biofilm-inhibiting effects observed in this study involved the inhibition of the initial stages of biofilm formation such as the adhesion of bacteria to the cell surface. Biofilm-producing bacteria such as *S. pneumoniae* adhere to host surfaces via Neu5Ac-containing glycans [32]. As *S. mutans* also adheres to surface glycans, SLs and Neu5Ac may competitively mask these glycan-binding sites of *S. mutans*, thereby inhibiting biofilm formation.

Interestingly, although 3′-SL and 6′-SL are structural isomers containing Neu5Ac, their physiological activities differed. Studies using recombinant enzymes have shown that HMO-degrading enzymes in *Bacteroides fragilis* and *Bifidobacterium* exhibit different reaction rates toward 3′-SL and 6′-SL [33,34]. The avian influenza virus recognizes Neu5Ac-containing glycans on host cells, and its infection-inhibition spectrum differs between 3′-SL and 6′-SL [35]. Our findings are consistent with these reports and suggest that HMOs inhibit biofilm formation by competitively binding to proteins involved in bacterial surface adhesion. The effect varied depending on the molecular structure of HMOs containing Neu5Ac. We found that SLs and Neu5Ac similarly inhibited biofilm formation across multiple *S. mutans* strains (Figure 1) and in mixed cultures of six *Streptococcus* species (Figure 2). These findings support the proposed mechanism of biofilm formation inhibition and partially explain how SL and Neu5Ac exert effects even in the presence of multiple bacterial species in the human oral cavity.

To further understand the mechanism by which HMOs suppress biofilm formation, we examined gene expression changes in *S. mutans* following HMO treatment. Compared with xylitol, 3′-SL increased the number of DEGs by twofold, whereas 6′-SL and Neu5Ac increased the number of DEGs by more than threefold. Conversely, 2′-FL was associated with approximately fivefold fewer DEGs than xylitol (Figure 4 and Figure S1). The large number of genes with expression changes aligned with the strong inhibition of biofilm formation in the crystal violet assay. This trend is consistent with reports investigating the effects of xylitol and carbohydrates on *S. mutans* biofilms [36]. Xylitol suppressed the expression of universal genes, such as ribosomal protein and histone acetyltransferases. In contrast, SLs and Neu5Ac downregulated genes involved in bacteriocin production and upregulated stress-responsive genes such as surfactin and bacitracin. The suppression of universal gene expression by xylitol may have resulted from the inhibition of biofilm formation, which prevents the growth and proliferation of *S. mutans*. SLs and Neu5Ac might have imposed more severe stress on *S. mutans* than xylitol, promoting the expression of stress-responsive genes. This suggested that SLs and Neu5Ac inhibited biofilm formation through physical adhesion inhibition and molecular stress. Furthermore, combining these agents with adjunctive therapies that have different mechanisms of action, such as photobiomodulation, may offer a more effective strategy for controlling biofilm formation.

This study had two limitations. First, the results were based on in vitro explorative experiments targeting only a small number of bacterial species. The human oral cavity hosts a diverse and complex bacterial flora, including *S. mutans* and other *Streptococcus* species, which form biofilms while cooperating or competing with one another [37,38]. Although we evaluated biofilm inhibition in mixed cultures of multiple *Streptococcus* species, this mixture represented only a part of the bacterial diversity present in the human oral cavity. HMO function should be further studied through ex vivo biofilm effect verification using human saliva. In addition, randomized clinical trials are necessary to more accurately evaluate the effects of HMOs. and randomized clinical trials. Another limitation was that the test compounds were used at higher concentrations than those reported in previous studies targeting *S. mutans* biofilms [27,39]. Human colostrum (human milk within 1 week after birth) contains the highest level of HMOs (up to approximately 15 g/L) [40]. HMOs practically exist at high concentrations in the oral cavities of newborns during lactation. Therefore, the concentration used in the present study is achievable in the oral cavity, and although it is limited, physiological significance could be expected. The physiological concentrations of HMOs in the human oral cavity under daily conditions remain unclear and should be clarified in future studies.

This is the first study to comprehensively elucidate the mechanisms by which 6′-SL and its substructure Neu5Ac inhibit biofilm formation in *S. mutans* and other *Streptococcus* species, and to highlight the importance of the molecular structure of HMOs containing Neu5Ac in this process. The null hypothesis was rejected. We found that 6′-SL inhibits *S. mutans* biofilm formation more effectively than its structural isomer 3′-SL and that Neu5Ac represents a core structure for the inhibitory effect of HMOs. Gene expression analysis suggested that the effect of the compounds involved physical inhibition of surface adhesion and gene expression regulation caused by stress. Moreover, the results suggested that SLs and Neu5Ac inhibited biofilm formation across multiple *Streptococcus* species. This study is expected to lead to the elucidation of the physiological significance of HMOs derived from human milk in the oral cavity of newborns. Furthermore, controlling oral biofilm formation using HMOs as functional foods for children after weaning and for adults may help reduce the risk of oral diseases and systemic diseases, thereby maintaining human health.

## 5. Patents

Y.S. has filed a provisional patent concerning the use of 6’-SL and Neu5Ac for the inhibition of bacterial biofilm formation.

## Figures and Tables

**Table 1:** Summary of *Streptococcus* biofilm quantification.

Group	*S. mutans* UA159			*S. mutans* 25175			*S. mutans* 35668			Mixture of *Streptococcus* Species		
	Absorbance (Mean ± SD)	*p* Value		Absorbance (Mean ± SD)	*p* Value		Absorbance (Mean ± SD)	*p* Value		Absorbance (Mean ± SD)	*p* Value	
		vs. NC	vs. Xylitol		vs. NC	vs. Xylitol		vs. NC	vs. Xylitol		vs. NC	vs. Xylitol
NC	2.10 ± 0.10	-	-	6.64 ± 0.34	-	-	5.47 ± 1.34	-	-	1.38 ± 0.32	-	-
Xylitol	1.25 ± 0.28	<0.01	-	4.23 ± 0.73	<0.01	-	2.63 ± 1.04	<0.01	-	1.29 ± 0.36	1.00	-
2′-FL	1.54 ± 0.12	<0.01	0.34	5.91 ± 0.66	<0.01	<0.01	2.64 ± 1.18	<0.01	1.00	1.07 ± 0.29	0.58	0.99
3′-SL	1.00 ± 0.09	<0.01	0.50	4.21 ± 0.64	<0.01	1.00	1.53 ± 0.66	<0.01	<0.01	0.78 ± 0.18	0.02	0.17
6′-SL	0.55 ± 0.06	<0.01	<0.01	3.81 ± 0.88	<0.01	0.20	0.89 ± 0.38	<0.01	<0.01	0.81 ± 0.05	0.03	0.23
Neu5Ac	0.40 ± 0.03	<0.01	<0.01	2.49 ± 0.30	<0.01	<0.01	0.81 ± 0.45	<0.01	<0.01	0.41 ± 0.06	<0.01	<0.01

NC = negative control, 3′-SL = 3′-sialyllactose, 6′-SL = 6′-sialyllactose, Neu5Ac = *N*-acetylneuraminic acid.

## Data Availability

The original contributions presented in this study are included in the article/Supplementary Material. Further inquiries can be directed to the corresponding author(s).

## Supplementary Materials

The following supporting information can be downloaded at: https://www.mdpi.com/article/10.3390/dj14010041/s1, Figure S1: Volcano plots of the differentially expressed genes on the *S. mutans* UA159 biofilm.

## Abbreviations

The following abbreviations are used in this manuscript:

SL	Sialyllactose
HMO	Human milk oligosaccharide
Neu5Ac	*N*-acetylneuraminic acid
2′-FL	2′-Fucosyllactose
3′-SL	3′-Sialyllactose
6′-SL	6′-Sialyllactose
BHI	Brain heart infusion broth
BHIs	BHI with 1% sucrose
OD_600_	Optical density at 600 nm
CLSM	Confocal laser-scanning microscopy
PI	Propidium iodide
RNA-Seq	RNA sequences
SD	Standard deviation
NC	Negative control
DEG(s)	Differentially expressed gene(s)
logFC	Log fold change
