# Supplementary material for: Inhibition of Streptococcus Biofilm Formation by 6′-Sialyllactose and N-Acetylneuraminic Acid
Source: Dent J (Basel). 2026 Jan 7;14(1):41. doi: 10.3390/dj14010041 (PMC12840428; doi:10.3390/dj14010041)
Supplement: Supplementary file 1 [file dentistry-14-00041-s001.zip › dentistry-3930799-supplementary.pdf]

Inhibition of *Streptococcus* Biofilm Formation by 6′-Sialyllactose and *N*-acetylneuraminic Acid

Yohei Sato, Yuta Watanabe, Tatsuhiro Ayabe, Takeshi Kokubo

Supplementary Materials

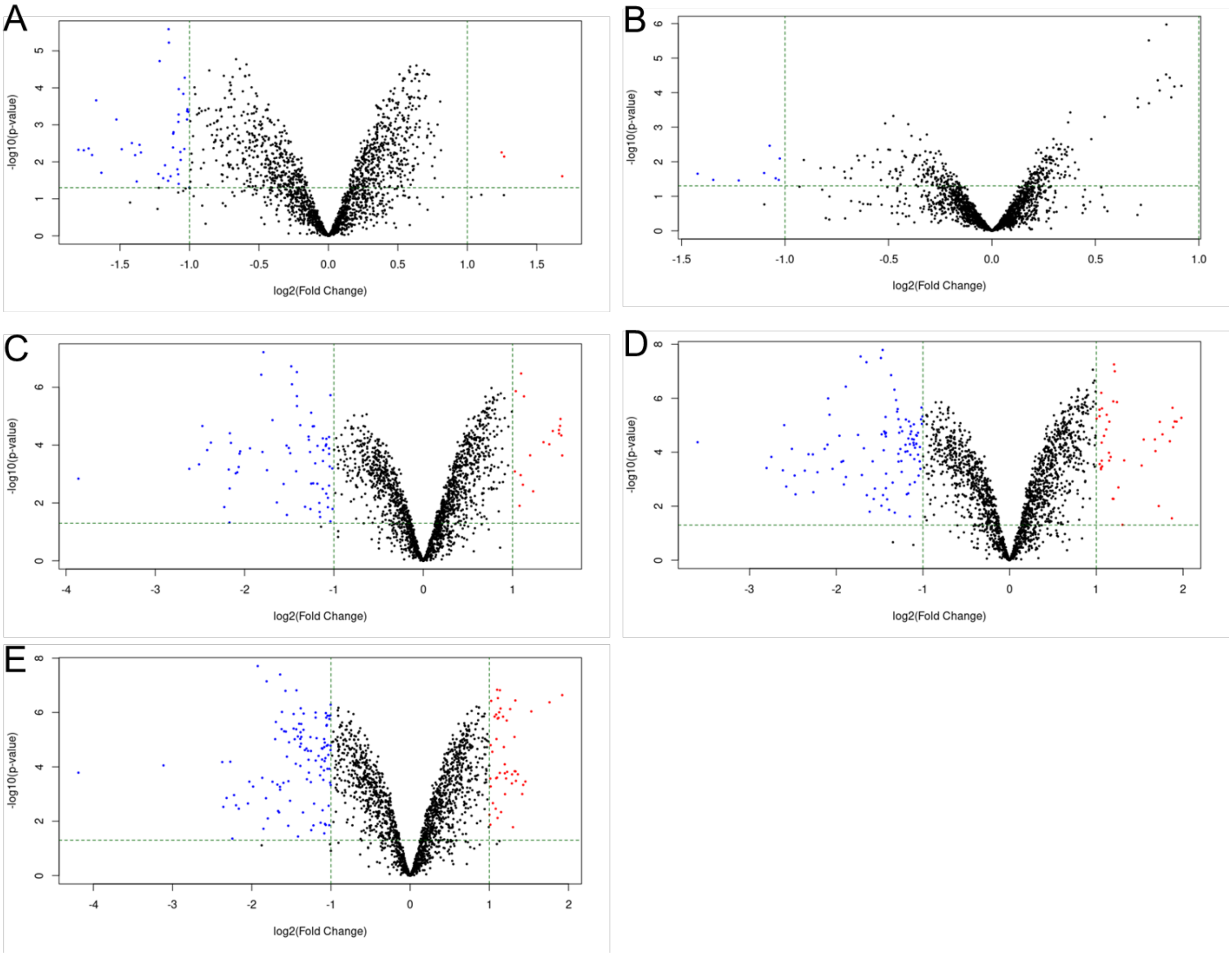

Figure S1. Volcano plots of the differentially expressed genes between NC and Xylitol (A), 2′-FL (B), 3′-SL (C), 6′-SL (D) and Neu5Ac (E) on the *S. mutans* UA159 biofilm.
